# A Dragon Fruit Picking Detection Method Based on YOLOv7 and PSP-Ellipse

**DOI:** 10.3390/s23083803

**Published:** 2023-04-07

**Authors:** Jialiang Zhou, Yueyue Zhang, Jinpeng Wang

**Affiliations:** 1 School of Mechanical and Electronic Engineering, Nanjing Forestry University, Nanjing 210037, China; 2 Co-Innovation Center of Efficient Processing and Utilization of Forest Resources, Nanjing Forestry University, Nanjing 210037, China

**Keywords:** deep learning, picking robot, YOLOv7, PSP-Ellipse, dragon fruit

## Abstract

Dragon fruit is one of the most popular fruits in China and Southeast Asia. It, however, is mainly picked manually, imposing high labor intensity on farmers. The hard branches and complex postures of dragon fruit make it difficult to achieve automated picking. For picking dragon fruits with diverse postures, this paper proposes a new dragon fruit detection method, not only to identify and locate the dragon fruit, but also to detect the endpoints that are at the head and root of the dragon fruit, which can provide more visual information for the dragon fruit picking robot. First, YOLOv7 is used to locate and classify the dragon fruit. Then, we propose a PSP-Ellipse method to further detect the endpoints of the dragon fruit, including dragon fruit segmentation via PSPNet, endpoints positioning via an ellipse fitting algorithm and endpoints classification via ResNet. To test the proposed method, some experiments are conducted. In dragon fruit detection, the precision, recall and average precision of YOLOv7 are 0.844, 0.924 and 0.932, respectively. YOLOv7 also performs better compared with some other models. In dragon fruit segmentation, the segmentation performance of PSPNet on dragon fruit is better than some other commonly used semantic segmentation models, with the segmentation precision, recall and mean intersection over union being 0.959, 0.943 and 0.906, respectively. In endpoints detection, the distance error and angle error of endpoints positioning based on ellipse fitting are 39.8 pixels and 4.3°, and the classification accuracy of endpoints based on ResNet is 0.92. The proposed PSP-Ellipse method makes a great improvement compared with two kinds of keypoint regression method based on ResNet and UNet. Orchard picking experiments verified that the method proposed in this paper is effective. The detection method proposed in this paper not only promotes the progress of the automatic picking of dragon fruit, but it also provides a reference for other fruit detection.

## 1. Introduction

The fruit picking robot is becoming one of the hottest topics in recent years [1,2]. China is the world’s second largest dragon fruit producer. Nowadays, dragon fruit is usually harvested manually by cutting its branch that is connected with the root, which causes a lot of labor intensity. Research is being conducted on the dragon fruit picking robot to realize automatic picking, which could greatly reduce the labor force [3,4]. Visual intelligence is of key importance to the fruit picking robot [5]. However, most of the research on the visual systems for fruit picking only focuses on the recognition and position of fruits, which is not enough for dragon fruits [6]. The branches of dragon fruit are very hard, which means that the only way to pick dragon fruit is to cut the branches together with the fruit. The difficulty in the automatic picking of dragon fruit is that the complex postures of dragon fruit make it difficult to accurately find the root position for cutting. Therefore, in order to realize automatic picking, we need to develop ways to detect the position of dragon fruit and its root position.

The common methods used for fruit detection are traditional machine vision-based methods and deep learning-based methods. The former needs to manually extract features to recognize fruits, and it is generally less robust which makes it difficult to apply in complex natural environments [7,8,9]. In recent years, deep learning-based object detection algorithms have been widely considered to be promising tools for fruit detection because of their better generalization ability, which may be grouped into two categories: the one-stage algorithm and the two-stage algorithm [10,11,12,13,14]. Normally, the inference speed of the two-stage algorithm is slower than that of the one-stage algorithm, but the two-stage algorithm has a higher accuracy. For a picking robot, picking efficiency and picking accuracy are both important. YOLO and RCNN are the representatives of the one-stage algorithm and two-stage algorithm, and many researchers have improved the inference speed and the detection accuracy based on these models [15,16,17]. Xu et al. [18] proposed an improved CBF module and replaced the bottleneck CSP module with the Specter module, which improved the detection accuracy and detection speed of the YOLOv5s model. Similarly, CIoU Loss function was used in training YOLOv5s to decrease the missed detection rate and false detection rate [19]. Yang et al. [20] introduced BCAM into the network and added BCAM between the backbone neck of the YOLOv5s model, which obtained a high detection accuracy. As for dragon fruit detection, some researchers have made relevant contributions in detection. For example, the backbone of YOLOv4 was replaced with Mobilenet-v3 to detect dragon fruit, thereby reducing the model size and improving speed. However, this method makes the average precision of the model decrease slightly [21]. Zhang et al. [22] proposed an improved YOLOv5s to accurately detect dragon fruits under different light conditions. These methods above just locate and classify the fruits, which is difficult to apply to picking just some fruits, such as dragon fruit.

As for automatic picking of some special fruits, some researchers have made some contributions. Liang et al. [23] use YOLOv3 to detect litchi, and then use UNet to separate the stem one by one. Qi et al. [24] proposed a method for identifying litchi picking position. They used YOLOv5 to detect the litchi main stem and used PSPNet to segment the main stem and obtained the pixel coordinates of picking points in the segmented image. Sun et al. [25] proposed a novel method to detect the keypoint on the branch. They first obtained a candidate area according to the fruit-growing position and the fruit stem keypoint detection and used a multi-level feature fusion network to further detect the keypoint. The methods mentioned above not only detect the fruit, but also detect the stems or branches for accurately picking. However, the main problem of these methods is that the methods are not new enough and the accuracy is low.

To address the difficulties in the automatic picking of dragon fruit due to its postural diversity, the goal of this work is to propose a method to classify dragon fruit according to its growth posture and detect the head and root position of dragon fruit to identify the growth direction and realize automatic picking. We have developed this method using an edge computing machine and built a dragon fruit picking system. Using the picking system, we also have conducted some picking experiments.

The main contributions of this paper are as follows:

1. For the first time, dragon fruits are classified into two categories according to their postures. YOLOv7 is used to locate and classify dragon fruits, and some hot models are compared.
2. We propose a PSP-Ellipse method for endpoints detection and compare the proposed method with two kinds of keypoint regression method.
3. We have built a set of dragon fruit picking systems. To a certain extent, the problem of the picking difficulty caused by the complex growth posture of dragon fruit has been solved from the perspective of vision.

## 2. Materials and Methods

### 2.1. Dataset Construction

The dragon fruit pictures used in this article were taken from orchard Lile agricultural, Nanjing city. The camera used for collecting images is MV-CE200-10UC. They were taken under different weather conditions (cloudy and sunny), day times (morning, noon, evening and night), lighting conditions (strong light, weak light and artificial light) and shooting distances. All these pictures under complex natural environments make the network model more robust. We randomly cropped, randomly rotated and brightness modified the original pictures for data augmentation, and 2458 pictures used for the object detection model were obtained after manually eliminating invalid pictures. Labelme software was used to manually mark the rectangular box area and generate label files.

In the subsequent picking experiments, the camera was fixed eye-to-hand and mounted on the base of the robotic arm. According to the postures of the dragon fruit in the camera view, this paper classifies dragon fruit into two categories. First, when a dragon fruit grows on the left or right side of its branch, it is named as the dragon fruit in the side (DF_S). This category of dragon fruit needs to detect its position, growth direction and endpoints position to provide a basis for the mechanical arm to pick. Second, when a dragon fruit grows on the front side of its branch, it is named as the dragon fruit in the front (DF_F). This category of dragon fruit can be picked by detecting its position directly.

By using some of the label files and original pictures, we cropped all of the dragon fruits named DF_S to train, validate and test the semantic segmentation model. Table 1 shows the datasets constructed.

**Figure 1:**
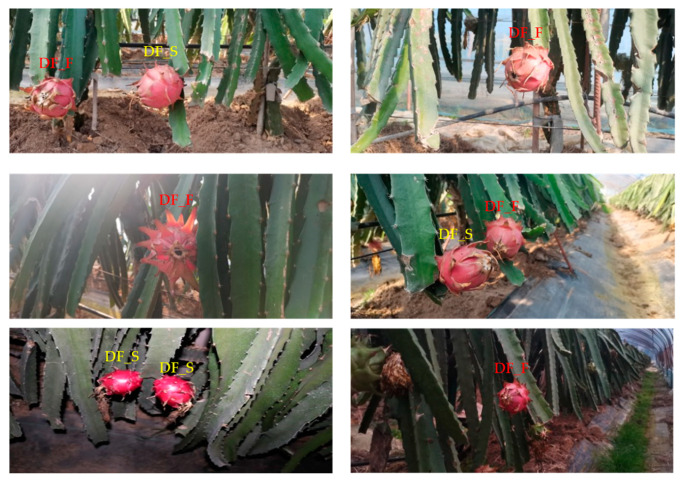
Data of two categories of dragon fruit taken at different times. DF_S denotes dragon fruits in the side, and DF_F denotes dragon fruits in the front.

Figure 1 shows some pictures in the dataset taken at different times and includes the two categories of dragon fruit.

### 2.2. Overview of the Method Proposed in this Paper

**Figure 2:**
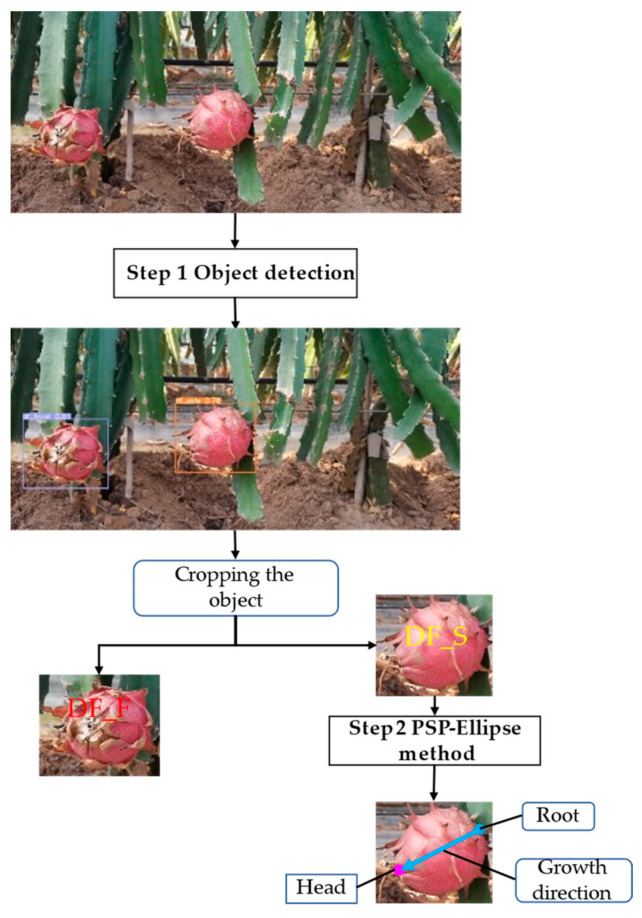
The flow chart of the proposed method.

The flow chart of the method proposed in this paper is shown in Figure 2, which can be divided into the following two steps.

(1) Step 1: The dragon fruit image is processed by the trained YOLOv7 network to locate the fruit objects and classify them into DF_S and DF_F, and the DF_S is cropped for further processing.
(2) Step 2: The cropped image is processed by the PSP-Ellipse method proposed in this paper to detect the head and root of the dragon fruit.

### 2.3. Methodology

#### 2.3.1. Related Theoretical Background

YOLO series models have been widely used in the agricultural field. YOLOv7 is currently the better performing object detector [26]. After pre-processing, the original image first enters the backbone network, which serves to extract features of several scales. Then, it enters the detection head to integrate the features extracted by the backbone network at several scales and perform feature fusion. Finally, the output of the YOLOv7 is obtained. In this paper, YOLOv7 is used for classifying the dragon fruit. The DF_S detected will further be processed.

PSPNet is an efficient semantic segmentation network that classifies each pixel of the input image [27]. The core parts of this network are the feature extraction network and the Pyramid Pooling Module. The input images first enter the backbone to obtain the feature (F_m). Some new features are obtained at different scales by pooling layers of different sizes; then, these features are upsampled separately to obtain the original scale features, and the newly obtained features are fused with the initial features. Finally, the segmented dragon fruit image is obtained by convolutional feature integration.

Ellipse fitting based on minimizing algebraic distance is a classic image processing algorithm to recognize the ellipse shape in the image. In the two-dimensional plane coordinate system, the ellipse can generally be expressed by Formula (1). The algebraic distance from a point on the plane to the elliptic curve is defined as shown in Formula (2). For a given point set, Formula (3) is to be solved.

(1) $$Ax^{2}+Bxy+Cy^{2}+Dx+Ey+F=0$$

(2) $$H_{i}=\left(x_{i}{}^{2},x_{i}y_{i},y_{i}{}^{2},x_{i}{,y}_{i},1\right)\;s=\left(A,B,C,D,E,F\right)\;D_{i}=H_{i}⊙s$$

(3) $$s*=Min\sum_{i=1}^{n}D_{i}{}^{2}$$

where *A*, *B*, *C*, *D*, *E* and *F* are coefficients of arbitrary elliptic equations, and *x* and *y* represent the coordinates of any point on the plane.

#### 2.3.2. PSP-Ellipse Method for Endpoints Detection

**Figure 3:**
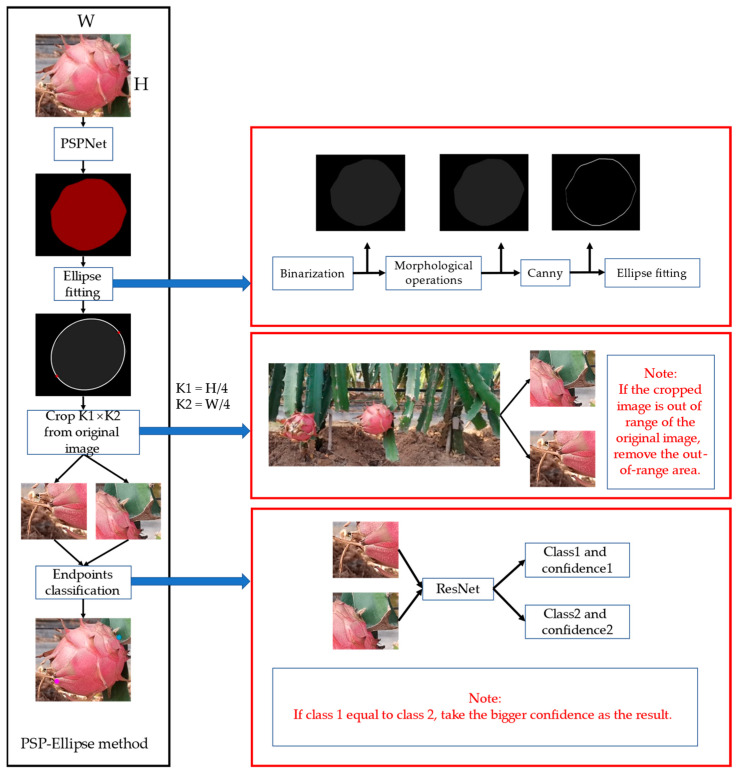
The black box is the flowchart of the PSP-Ellipse method. The red box is a detailed description of each step. The red font is a note for this operation.

The PSP-Ellipse method can be divided into three steps. First, the cropped images from YOLOv7 first enter the PSPNet to produce a segmented image. Second, the segmented image is processed by binarization, morphological operations, edges extraction and ellipse fitting. We use the fitted ellipse to calculate the position of the two endpoints of the long axis, and we regard the two endpoints as the endpoints of the dragon fruit. Third, taking the two endpoints obtained by ellipse fitting as the center, we crop two K1 × K2 rectangular areas from the original image and classify the rectangular image with a trained convolution neural network, which can directly output whether a point is the head or the root. K1 and K2 are calculated according to the size of the cropped dragon fruit image. Figure 3 shows the schematic of the PSP-Ellipse method.

**Figure 4:**
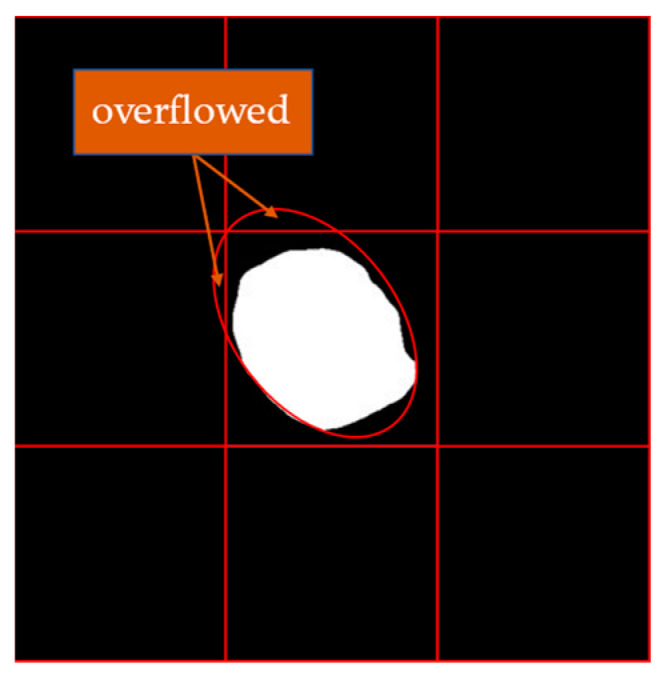
The schematic of filling pixels.

We have found two potential problems in the second step. First, the ellipse fitted by a small number of images will exceed the size of the segmented image and influence the subsequent calculations. Therefore, we will fill the surrounding of the segmented image with 0 value pixels as shown in Figure 4. Second, it was found that some noise points may influence ellipse fitting. Therefore, for all the ellipses fitted, we will calculate their IoU with the dragon fruit area; then, the ellipse with the largest IoU with the dragon fruit area is the fitting result.

In the third step, there are some details to handle. When the fruit appears near the edge of the original image, one of the rectangular areas may exceed the image range. For this, we will remove the parts beyond the image range. In addition, when ResNet processes two rectangular area images with the same output results, we believe that the one with a higher prediction confidence is correct.

### 2.4. Experimental Environment and Training Strategies

The training environment for the YOLOv7 network and PSPNet is shown as follows: the operation system is Windows 10, the GPU is NVIDIA GeForce RTX 3060Ti, the CPU is AMD Ryzen 5 3600X and the deep learning framework is Pytorch.

For the training of the YOLOv7 network, we use mosaic and mixup for data enhancement, and the probabilities are 1.0 and 0.15. In label assignment, the intersection over union (IoU) threshold is 0.2, the aspect ratio threshold of the anchor is 4.0. Other hyperparameters used are as follows: the training epoch is 300, the training image size is 640 × 640, the initial learning rate is 0.01 and the parameters in convolution kernel are initialized randomly according to the Gaussian distribution. The training ultimately saves the weight file with the least loss on the validation set.

For the training of PSPNet, the hyperparameters used for training are shown as follows: the input image size is 224 × 224, the training epoch is 100, the batch size is 2, the weight decay coefficient is 10-4, the initial learning rate is 10-2, the learning rate decay type is cosine function, the minimum learning rate is 10-4, the optimizer is SGD and the momentum is 0.9. In this paper, the number of pixels in the background and the number of dragon fruit pixels may be unbalanced, so dice loss is used to reduce the effect of an unbalanced sample size. The training ultimately saves the weight file with the least loss on the validation set.

## 3. Results

All of the evaluation metrics used in this paper are described as follows.

Precision (P), recall (R), average precision (AP) and mean average precision (mAP) are used in the test experiments of YOLOv7. P, R, intersection over union (IoU) and mean intersection over union (MIoU) are used in the test experiments of PSPNet. Distance error (DE), angle error (AE) and classification accuracy (CA) are used in the test experiments of PSP-Ellipse. They are calculated as follows:

(4) $$P=\frac{TP}{TP+FP}$$

(5) $$R=\frac{TP}{TP+FN}$$

(6) $$AP=\int_{0}^{1}P(R)dR$$

(7) $$mAP=\frac{\sum_{i=1}^{n}AP_{i}}{n}$$

(8) $$IoU=\frac{TP}{TP+FP+FN}$$

(9) $$MIoU=\frac{\sum_{i=0}^{k}IoU_{i}}{k+1}$$

(10) $$DE=\frac{\sqrt{{\left({\hat{x}}_{h}-x_{h}\right)}^{2}+{\left({\hat{y}}_{h}-y_{h}\right)}^{2}}+\sqrt{{\left({\hat{x}}_{r}-x_{r}\right)}^{2}+{\left({\hat{y}}_{r}-y_{r}\right)}^{2}}}{2}$$

(11) $$AE=\left|\arctan\frac{\left(y_{h}-y_{r}\right)}{\left(x_{h}-x_{r}\right)}-\arctan\frac{\left({\hat{y}}_{h}-{\hat{y}}_{r}\right)}{\left({\hat{x}}_{h}-{\hat{x}}_{r}\right)}\right|$$

(12) $$CA=\frac{T}{T+F}$$

where *TP* is the number of positive samples predicted to be positive class; *FP* is the number of negative samples predicted to be positive class; *FN* is the number of positive samples predicted to be negative class; *AP* is the area below the PR curve; *mAP* is the mean *AP* value for each category; and *n* and *k* represent the number of categories in object detection and semantic segmentation. In this paper, *n* = 2 and *k* = 1, and *x* and *y* are horizontal and vertical coordinates of endpoints. The subscripts *h* and *r* represent the head and root, respectively. The superscript means it is ground truth; the other is the predicted results. *T* and *F* are the number of truly classified samples and falsely classified samples in endpoints classification.

### 3.1. Training and Test Results of YOLOv7

**Figure 5:**
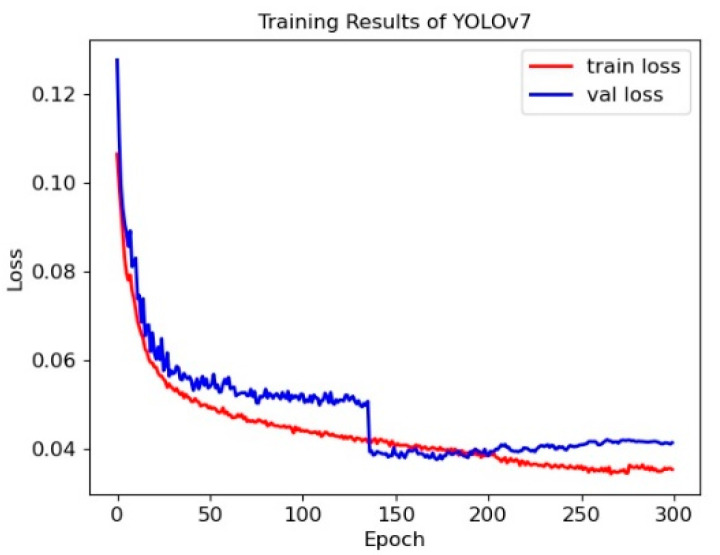
Training results of the YOLOv7 network.

Using the dataset in Table 1, we train the YOLOv7 network. The loss of the training process is shown in Figure 5.

As shown in Figure 5, the loss in the training set starts to drop faster and almost stops dropping later. The loss of the validation set starts to drop faster and drops sharply when the training reaches 136 epochs, indicating that the model jumps out of the local optimal point and almost stops dropping at the later epoch.

To verify that the trained model has better robustness, the model is tested. The number of test images is 296, the number of labels for DF_S and DF_F is 245 and 212, respectively, the IoU threshold for non-maximum suppression is 0.5, the input image size is 640 × 640, and the batch size is 32. Table 2 shows the test results.

As shown in Table 2, the precision of the model is significantly lower than the recall, which means that the classification error of the model is large. A possible reason for this is that part of the fruit posture is between the two categories, which is difficult to strictly distinguish. We also found that the model performs better in detecting DF_F due to more samples of DF_F in training.

To further verify that the object detector used in this paper is superior, it is compared with other commonly used object detectors, and the comparison results are shown in Table 3.

Comparative experimental results show that the accuracy of YOLOv7 is higher than some early networks. Compared with SSD, YOLOv4, YOLOv5, YOLOX and YOLOv6, the precision of YOLOv7 was improved by 0.113, 0.182, 0.077, 0.049 and 0.065, the recall was improved by 0.064, 0.161, 0.076, 0.042 and 0.056, and the average precision was improved by 0.107, 0.301, 0.093, 0.053 and 0.085.

### 3.2. Training and Test Results of PSPNet

**Figure 6:**
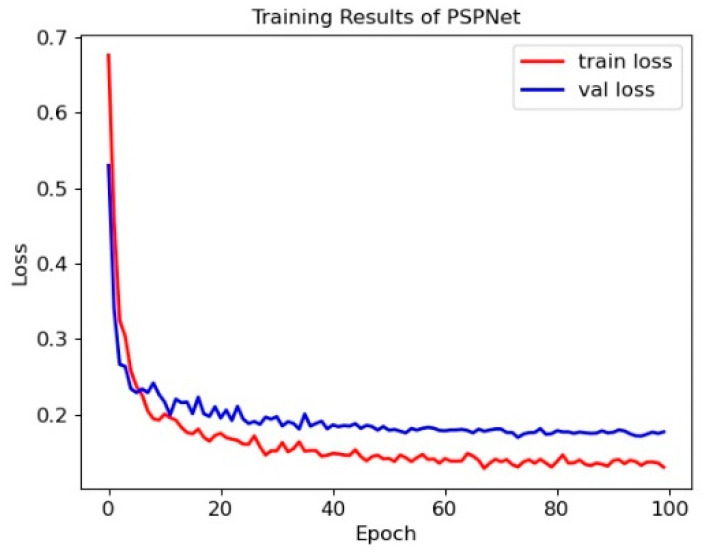
Training results of the PSPNet.

Using the dataset in Table 1, we train the PSPNet. The loss of the training process is shown in Figure 6.

As shown in Figure 6, the loss in the training set and validation set start to drop faster and almost stop dropping later.

To verify that the trained model has better robustness, the model is tested. The number of test images is 146, the input image size is 224 × 224, and the batch size is 10. Table 4 shows the test results.

As shown in Table 4, we pay more attention to the performance of the model in segmenting dragon fruit. All the precision, recall and IoU reach a very high value due to the dice loss in training.

We also compared the performance of PSPNet with other commonly used semantic segmentation models, and the results are shown in Table 5.

**Figure 7:**
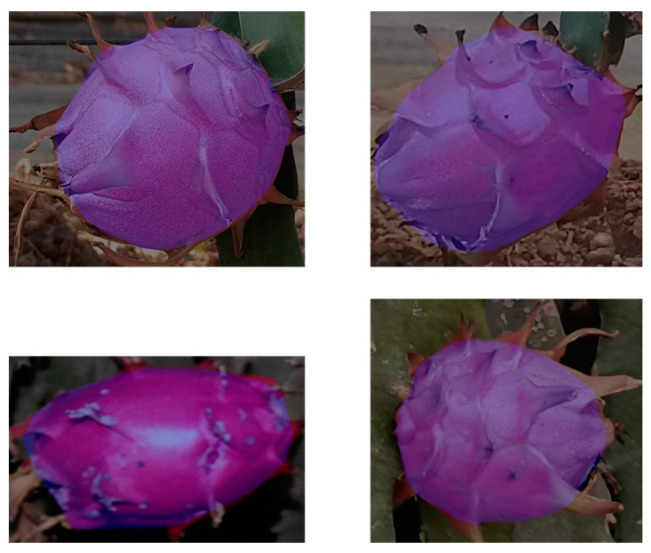
The mixing results of the image segmented by PSPNet and the dragon fruit image.

Comparative experimental results show that the accuracy of PSPNet is higher than other commonly used semantic segmentation networks. Compared with FCN, DeepLabv3, LR-ASPP and UNet, the precision of PSPNet was improved by 0.03, 0.038, 0.045 and 0.021, the recall was improved by 0.039, 0.05, 0.072 and 0.026, and the mean intersection over union was improved by 0.021, 0.063, 0.104 and 0.014. Figure 7 shows the mixing results of the image segmented by PSPNet and the dragon fruit image.

### 3.3. Endpoints Detection Results of PSP-Ellipse Method

The head and root have different features. For example, there may be flowers near the head, and the shape of the fruit near the head may be different from the shape of the fruit near the root. There are always branches near the root. Convolutional neural networks can learn these features themselves and distinguish between the head and the root through these features. Based on this, we use ResNet to classify endpoints, which means that ResNet directly outputs classification results after processing the two rectangular area images. Some other classification models are also compared with ResNet to verify its effectiveness. The classification models are trained by using some cropped rectangular images near the two endpoints. To verify the effectiveness of the PSP-Ellipse method proposed in this paper, the end-to-end test is carried out. We use 200 ground truth images which are cropped using YOLOv7 for the test, and these images are manually labeled the endpoints position.

Keypoint regression is also a method for detecting the endpoints of dragon fruit. We conducted two kinds of keypoint regression method. The first is direct regression. This method adds a fully connected layer to the end of a convolutional neural network, and finally outputs four values, which correspond to the head and root coordinates. The second is based on heatmaps. This method is to make a circular heatmap with the keypoint as the center. The closer to the center point, the larger the value, and the further away from the center point, the smaller the value. Therefore, we trained the ResNet and UNet for the two kinds of keypoint regression method as comparative experiments. The results are shown in Table 6.

**Figure 8:**
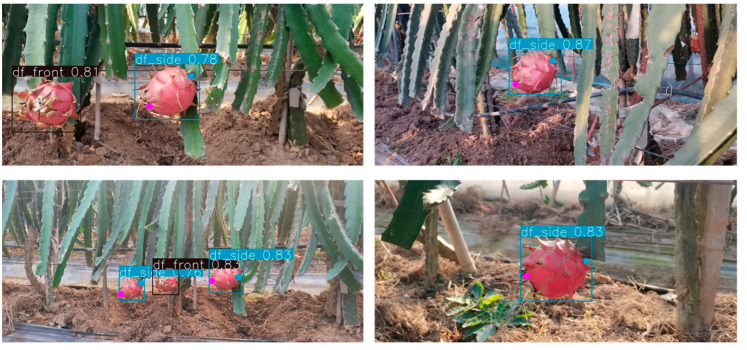
Dragon fruit detection results of the method proposed in this paper. df_front denotes DF_F and df_side denotes DF_S.

As shown in Table 6, using ResNet for endpoints classification has a higher accuracy. Compared with LeNet, VGGNet and MobileNet, the classification accuracy improves by 0.05, 0.02 and 0.03. In addition, the PSP-Ellipse method for endpoints detection obtains a great improvement compared with the keypoint regression method. As for an original image of size 4608 × 2064, using the PSP-Ellipse method proposed in this paper, the distance error and angle error are only 39.8 pixels and 4.3°. Compared with the two kinds of keypoint regression method, the distance error is decreased by 65.2% and 51.1%, and the angle error is decreased by 70.5% and 47.6%. At the same time, the classification accuracy is increased by 119.0% and 76.9%. The experimental results fully demonstrate that the PSP-Ellipse method proposed in this paper is very meaningful for detecting the endpoints of dragon fruit. Figure 8 shows some detection results of PSP-Ellipse. The blue point represents the position of the root, and the red point represents the position of the head.

### 3.4. Orchard Picking Experiment

**Figure 9:**
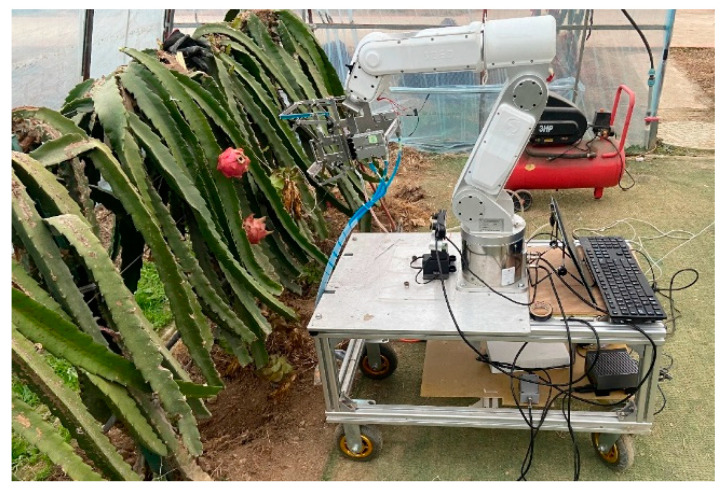
Dragon fruit picking experiment scene in orchard.

In order to verify that the method proposed in this paper can be used to pick dragon fruit, we conducted an orchard picking experiment. The picking robot is shown in Figure 9. The edge computing machine is Jetson AGX Orin 32GB, the depth camera is ZED Mini and the mechanical arm is S6H4D_Plus.

**Figure 10:**
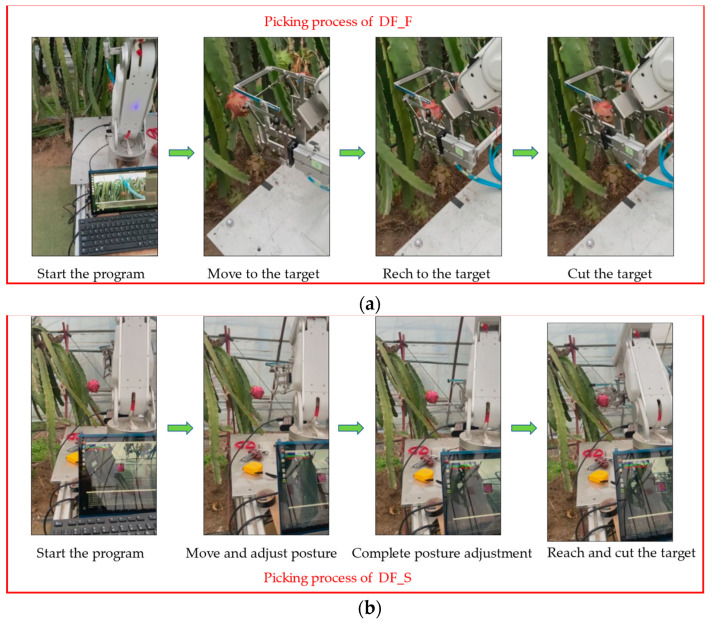
Picking process of the two categories of dragon fruit. (**a**) Picking process of DF_F; (**b**) picking process of DF_S.

For the two classifications of dragon fruit, the picking strategy is slightly different. For DF_F, directly send the three-coordinate command of moving to the center point to the mechanical arm. For DF_S, we need to calculate its growth direction and posture according to the endpoints found by the method proposed in this paper; then, control the mechanical arm to approach the fruit in a specific posture to achieve picking. Figure 10 shows the picking process of the two-classification dragon fruit.

## 4. Conclusions

In order to solve the problem of picking difficulties caused by the complex growth of dragon fruit, this paper proposes a method for dragon fruit detection, including fruit detection and endpoints detection, to enable a picking robot’s visual system to locate and determine the posture of dragon fruit. YOLOv7 is used for locating the dragon fruit and classifying dragon fruit into DF_S and DF_F. PSP-Ellipse is proposed for endpoints detection.

Several technological findings are made regarding the performances between competing algorithms along the two key steps of dragon fruit detection and endpoints detection. In dragon fruit detection, the precision, recall and average precision of YOLOv7 are 0.844, 0.924, 0.932, respectively; YOLOv7 therefore outperforms fellow object detection models in all metrics, including SSD, YOLOv4, YOLOv5, YOLOX and YOLOv6. In dragon fruit segmentation, the precision, recall and mean intersection over union of PSPNet are 0.959, 0.943, 0.906, respectively. PSPNet therefore also outperforms other commonly used models. In endpoints detection, the distance error, angle error and classification accuracy of PSP-Ellipse are 39.8 pixels, 4.3° and 0.92. Compared with the two kinds of keypoint regression method, the distance error is decreased by 65.2% and 51.1%, the angle error is decreased by 70.5% and 47.6%, and the classification accuracy increased by 119.0% and 76.9%. We built a dragon fruit picking system using the method proposed in this paper and conducted orchard picking experiments. We succeeded to pick some dragon fruit with different postures which further proves that the method proposed in this paper is effective.

## Figures and Tables

**Table 1:** The dataset for object detection and semantic segmentation.

Dataset	Training	Validation	Test
Object detection	1966	196	296
Semantic segmentation	1279	143	146

**Table 2:** Test results of YOLOv7.

Class	P	R	AP
DF_S	0.819	0.905	0.914
DF_F	0.870	0.943	0.95
Average	0.844	0.924	0.932

**Table 3:** Comparison results of YOLOv7 and other networks.

Networks	P	R	mAP
SSD	0.731	0.860	0.825
YOLOv4	0.662	0.763	0.631
YOLOv5	0.767	0.848	0.839
YOLOX	0.795	0.882	0.879
YOLOv6 [28]	0.779	0.868	0.847
YOLOv7	0.844	0.924	0.932

**Table 4:** Test results of PSPNet.

Class	P	R	IoU
Background	0.967	0.904	0.870
Dragon fruit	0.951	0.982	0.942
Average	0.959	0.943	0.906

**Table 5:** Comparison results of PSPNet and other networks.

Networks	P	R	MIoU
FCN	0.929	0.904	0.885
DeepLabv3	0.921	0.893	0.843
LR-ASPP	0.914	0.871	0.802
UNet	0.938	0.917	0.892
PSPNet	0.959	0.943	0.906

**Table 6:** Comparison results of several endpoint detection methods.

Methods	DE (Pixels)	AE (°)	CA
PSP-Ellipse-ResNet	39.8	4.3	0.92
PSP-Ellipse-LeNet	39.8	4.3	0.87
PSP-Ellipse-VGGNet	39.8	4.3	0.90
PSP-Ellipse-MobileNet	39.8	4.3	0.89
Direct regression-ResNet	114.5	14.6	0.42
Heatmap-UNet	81.4	8.2	0.52

PSP-Ellipse-ResNet refers to the PSP-Ellipse method using ResNet for endpoints classification, PSP-Ellipse-LeNet refers to the PSP-Ellipse method using LeNet for endpoints classification, PSP-Ellipse-VGGNet refers to the PSP-Ellipse method using VGGNet for endpoints classification, PSP-Ellipse-MobileNet refers to the PSP-Ellipse method using MobileNet for endpoints classification, Direct regression-ResNet refers to the direct endpoints regression method using ResNet, and Heatmap-UNet refers to the endpoints regression based on the heatmap using UNet.

## Data Availability

The data that support the findings of this study are available on request from the corresponding author.
